# Ectopic hepatocellular carcinoma manifesting multiple abdominal masses: A case report

**DOI:** 10.1097/MD.0000000000008968

**Published:** 2017-12-01

**Authors:** RenAn Jin, Qingsong Yu, Xiao Liang

**Affiliations:** a Department of General Surgery, Institute of Minimally Invasive Surgery, Sir Run Run Shaw Hospital, School of Medicine, Zhejiang University, Hangzhou; b Department of General Surgery, Shengzhou City People's Hospital, Shengzhou, China.

**Keywords:** abdominal mass, alpha-fetoprotein, ectopic liver, hepatocellular carcinoma

## Abstract

**Rationale:**

Ectopic hepatocellular carcinoma (HCC) is a rare disease that mostly originates from an ectopic liver.

**Patient concerns:**

The patient was admitted with upper abdominal distention for 3 months, which aggravated after meal.

**Diagnoses:**

A contrast-enhanced computed tomography (CT) scan revealed multiple abdominal masses. After exploratory laparotomy and histological examination, the patient was diagnosed as ectopic HCC.

**Interventions:**

Exploratory laparotomy was performed for the purpose of diagnosis and treatment.

**Outcomes:**

The tumors were excised by surgery and his symptom of upper abdominal distention was disappeared. A second surgery was performed for tumor recurrence and the patient died with total survival time of 22 months.

**Lessons:**

Ectopic HCC was usually in clinically silent, unless compression symptoms or intra-abdominal bleeding appeared. It did not have any typical character features in CT or Magnetic resonance imaging, may present with multiple abdominal masses. Surgery resection seems to be one of the effective treatments for ectopic HCC, though it is detected with multiple tumors.

## 1 Introduction

Ectopic hepatocellular carcinoma (HCC) is defined as HCC arising from hepatic parenchyma located in an extrahepatic organ or tissue. It mostly originates from an ectopic liver that is considered as a rare developmental error due to early or late developmental anomalies from hepatic tissue migrating aberrantly within the septum transversum or onto the pars cystic.^[1]^ Thus, ectopic HCC is even rarer. We report a case of ectopic HCC manifesting multiple abdominal masses located in different positions. To the best of our knowledge, there have not any similar cases reported before.

## 2 Case report

A 56-year-old man was admitted to our hospital for further evaluation of multiple abdominal masses. He had felt upper abdominal distention for 3 months, which aggravated after meal. There were no other associated symptoms. The patient was a chronic smoker without any other special hobbies, and denied hepatitis. Informed consent for study was obtained from the patient.

Physical examination found nothing unusual. The laboratory tests, including complete blood count and liver function, were almost in the normal range. Hepatitis B virus surface antigen (HBs Ag) and hepatitis C virus antibody (HCV Ab) were seronegative, hepatitis B virus surface antibody (HBs Ab) was positive, hepatitis B virus DNA was <1000 copies/mL. Serum level of carbohydrate antigen 125 (CA-125) and carcinoembryonic antigen (CEA) were slightly elevated (CA-125 148.50 U/mL, CEA 6.42 ng/mL), and the serum alpha-fetoprotein (AFP) was in the normal level (8.03 ng/mL).

**Figure 1:**
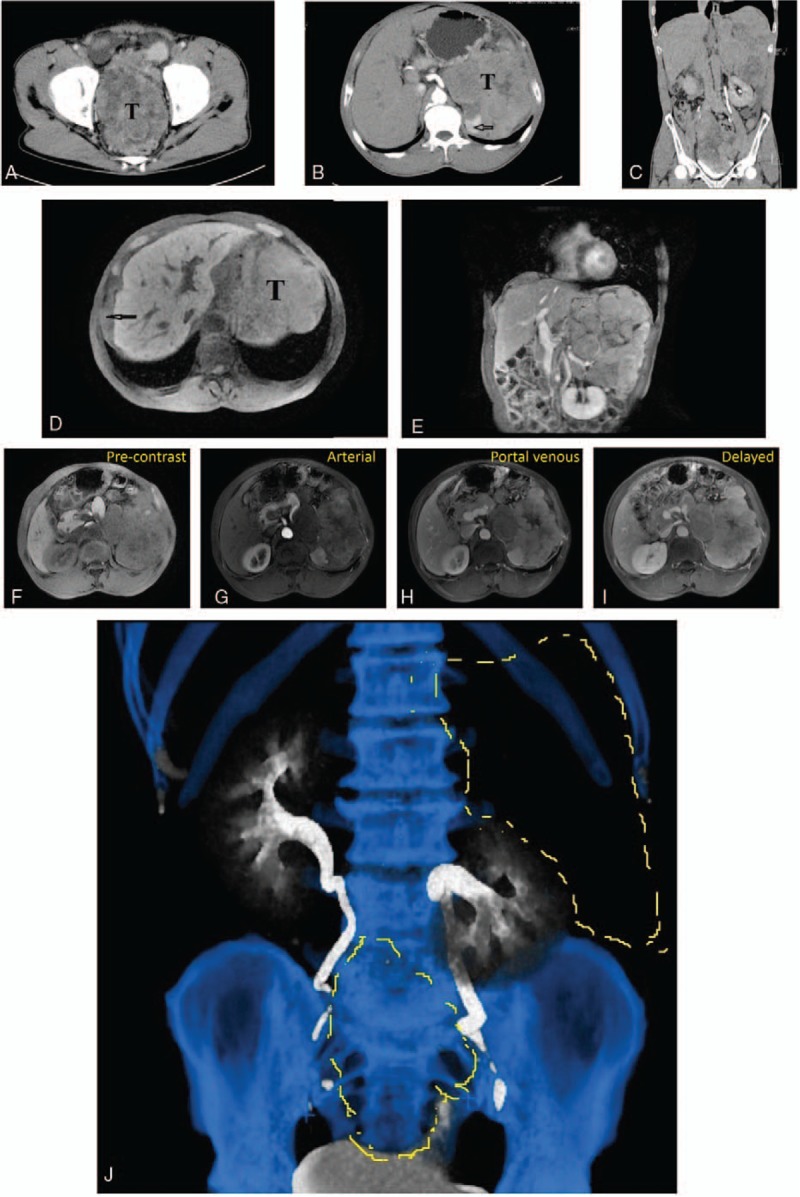
(A–C) Contrast-enhanced computed tomography of the entire abdomen found multiple masses located in left upper quadrant, hepatogastric gap, and pelvic cavity with irregular margin, heterogeneous density, and significant enhancement, involved to the spleen (arrow in B) and bladder. (D, E) Magnetic resonance imaging (MRI) of upper abdomen revealed a huge tumor (T) compressed the left kidney in the left upper quadrant without any communication with left liver lobe, another sharp edged soft tissue mass (arrow) located between the right liver and diaphragm. (F–I) MRI results showed masses presented with heterogeneous hyperenhancement in arterial phase, and more obvious hyperenhancement can be seen in portal venous and delayed phase, no washout of the contrast agent was detected. (J) CT urography revealed pressure on the bottom of the left ureter and left kidney.

A contrast-enhanced computed tomography (CT) scan revealed multiple masses located in left upper quadrant, hepatogastric gap, right abdomen, and excavatiorectovesicalis with irregular margin, heterogeneous density, and significant enhancement. The spleen, bladder, prostate, and liver capsule were seemed to be involved (Fig. 1A–C). Magnetic resonance imaging (MRI) also confirmed these findings (Fig. 1D, E), these masses presented with heterogeneous hyperenhancement in arterial phase, and more obvious hyperenhancement can be seen in portal venous and delayed phase, no washout of the contrast agent was detected (Fig. 1F–I). Fat tissues were detected inside the masses both by CT and MRI. There was no remarkable abnormality in the liver shown on CT or MRI. CT urography revealed pressure on the bottom of the left ureter and left kidney (Fig. 1J).

**Figure 2:**
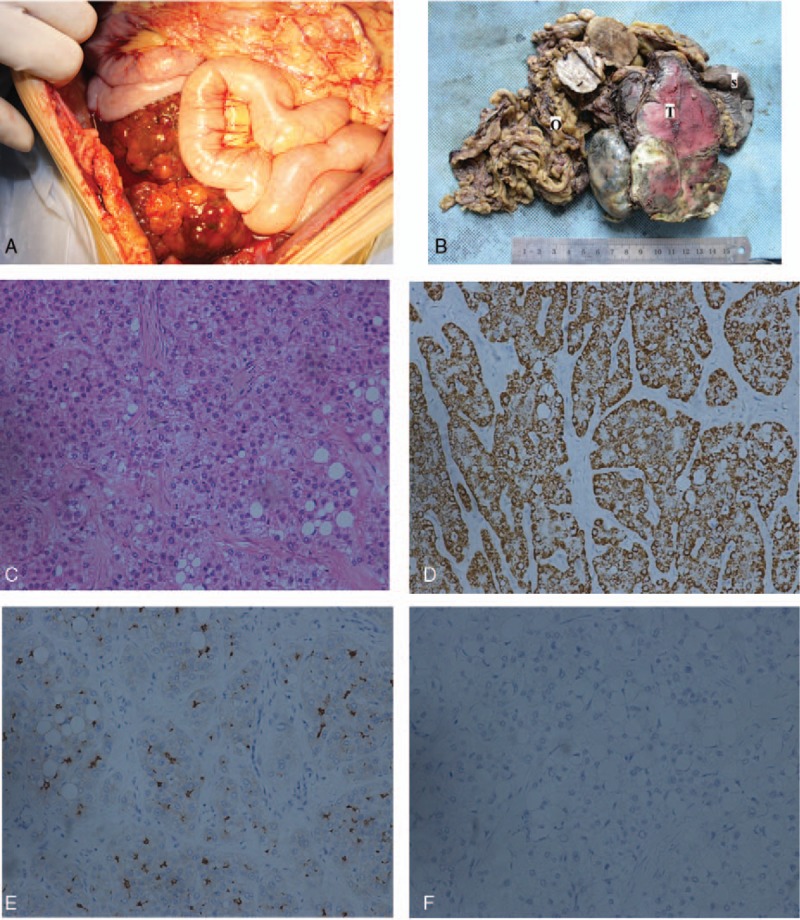
(A) Mass in the pelvic cavity without any adhesions to connective tissues. (B) Grossly specimens (part) consist of a well-demarcated, solid firm mass (T) with hemorrhagic and necrotic foci, spleen (S), and omentum (O). The mass was composed of several small and large nodules. (C) The tumor cells distributed in cords and trabecula, lots of fat cells were also seen (hematoxylin–eosin stain × 200). (D) The tumor cells were positive for hepatocyte paraffin 1 monoclonal antibody (hep par1 stain × 200). (E) The tumor cells were positive for CEA (CEA stain × 200). (F) The tumor cells were negative for AFP (AFP stain × 200).

Exploratory laparotomy was performed for the purpose of diagnosis and treatment. Intraoperatively, a giant irregular friable mass was located in upper abdomen, measured 30 cm × 20 cm × 15 cm, adhered to the spleen, omentum, and left diaphragm, blood supplied by vessel derived from splenic vessel, and no connection was identified between the tumor and the edge of the left hepatic lobe. Another mass was in pelvic cavity, 20 cm × 15 cm × 10 cm in size, had no adhesions to connective tissues (Fig. 2A). There were several small nodules seen on omentum and right diaphragm. There was no remarkable abnormality found in the liver. A fast frozen pathology suggested a diagnosis of malignant tumor; liposarcoma was first considered. All the tumors were excised besides spleen and omentum. His symptom of upper abdominal distention was disappeared. The postoperative period was uneventful.

Grossly, specimen consisted of well-demarcated, solid firm masses, spleen, omentum. The cut surface of the mass was bright yellow, and had a focal hemorrhagic and necrotic appearance. Although the tumor adhered to the spleen, it had no invasion to the spleen (Fig. 2B). Histological examination showed that the tumor was composed of proliferating large ovoid polygonal cells distributed in cords and trabeculae, with large and dark nuclei and abundant, eosinophilic cytoplasm (Fig. 2C). Immunohistochemical analysis performed on formalin-fixed paraffin-embedded sections showed that the tumor cells were positive for hepatocyte (Fig. 2D), CEA (Fig. 2E), and cytokine (CK) 8, but negative for AFP (Fig. 2F), CK-Pan, CK7, VM, S100, and HMB45. According to these findings, it confirmed the hepatocellular nature of the tumor. However, no noncancerous liver tissue was identified in any of the masses.

**Figure 3:**
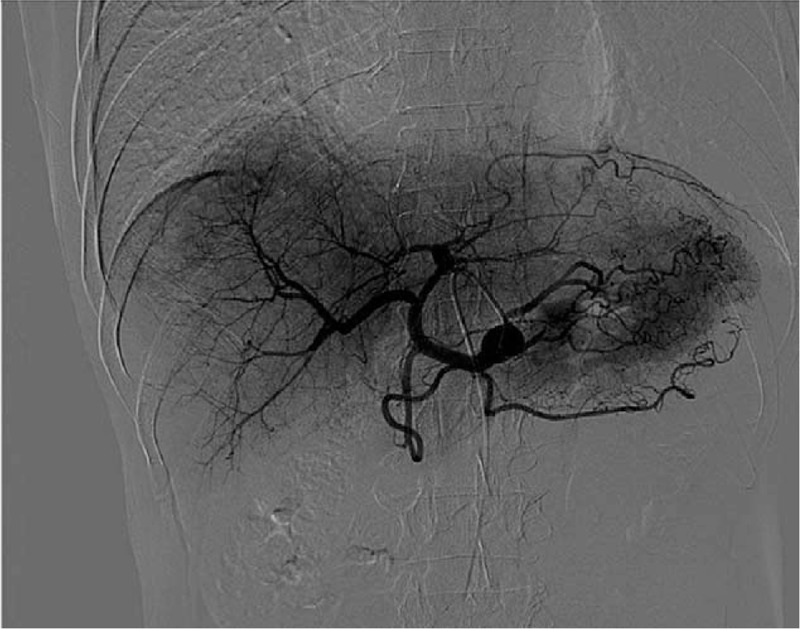
Digital subtraction angiography. Found nothing abnormal in the mother liver.

**Figure 4:**
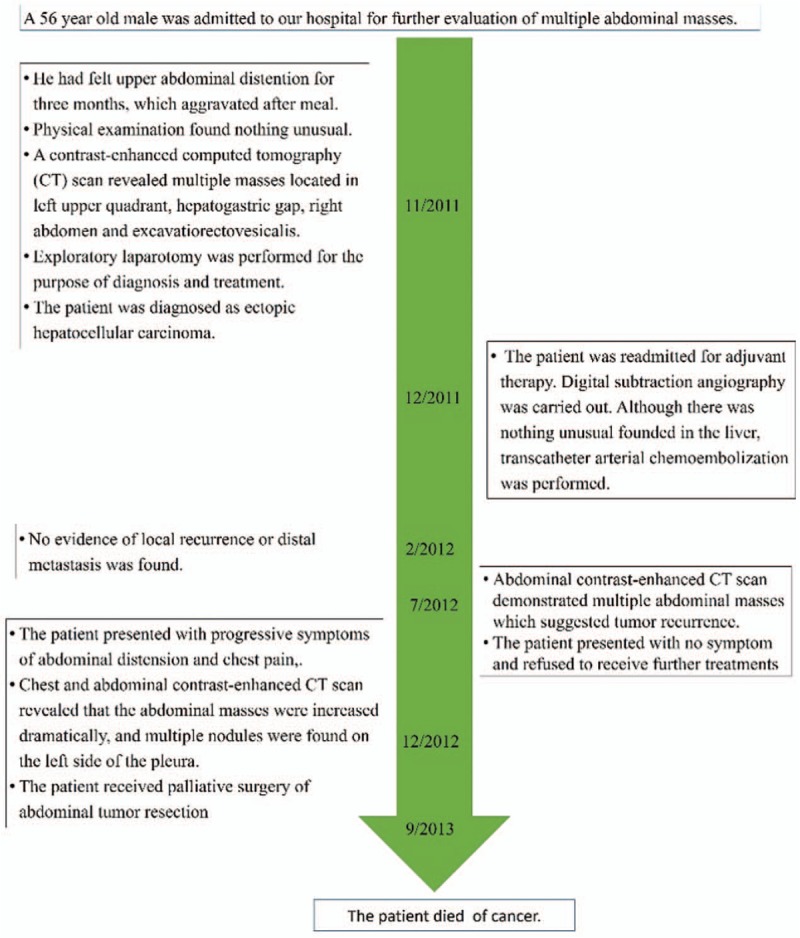
Timeline of this case.

One month after operation, the patient was readmitted for adjuvant therapy. The level of CA-125 and CEA was decreased to normal range. Digital subtraction angiography (DSA; Fig. 3) was carried out. Although there was nothing unusual founded in the liver; transcatheter arterial chemoembolization was performed. The patient remains well with no evidence of local recurrence or distal metastasis followed up for 3 months. Eight months after operation, abdominal contrast-enhanced CT scan demonstrated multiple abdominal masses that suggested tumor recurrence. The patient presented with no symptom at that time and he refused to receive further treatments. Thirteen months after operation, the patient presented with progressive symptoms of abdominal distension and chest pain; chest and abdominal contrast-enhanced CT scan revealed that the abdominal masses were increased dramatically; and multiple nodules were found on the left side of the pleura. The patient received palliative surgery of abdominal tumor resection. The symptom of abdominal distension was alleviated after surgery and he received effective pain control to improve the quality of life. Nine months after the second surgery, the patient died, with total survival time of 22 months. The timeline can be seen in Figure 4.

## 3 Discussion

The final diagnosis in our patient was primary ectopic HCC. The origin of ectopic HCC is still unknown, and it is mostly considered to be developed in ectopic liver tissue that is defined as liver tissue distinctly outside the mother liver without any communication with the mother liver.^[2]^ Ectopic liver can occur in various locations both in abdomen and thorax, more common in areas around the liver.^[3]^

As the ectopic liver with or without carcinogenesis is usually in clinically silent, it will not be easy to find unless compression symptoms or intra-abdominal bleeding appeared. Our patient went to see doctor for abdominal distention, 3 months before that he felt nothing uncomfortable. It did not have any typical character features in CT or MRI.

Ectopic liver seems to be more prone to hepatocarcinogenesis. A review of literature suggested that approximately 100 cases of ectopic liver had been reported and HCC was detected in 28 cases up to 2011.^[4]^ In our patient, the chronic hepatitis virus markers, HBs Ag and anti-HCV antibody, were negative, and the mother liver had no cirrhosis. According to Arakawa et al,^[5]^ the hepatitis B surface antigen was positive only in 1 (total 14 tested) case; the liver was cirrhotic in only 6 of 22 cases (27%). Thus, we speculate that the virus infection, whose role was emphasized in the sequence viral hepatitis → liver cirrhosis→ hepatocarcinogenesis, was not essential for ectopic liver developing into HCC. As ectopic liver tissue is not always as fully developed as the mother liver, in our case, the tumor was supplied by an artery not derived from the hepatic artery without the portal vein system, and the bile duct system was not found even examined under a microscope. It is theorized that due to the structure defect, the foci of ectopic liver tissue may be metabolically handicapped, leading to longer exposure to various carcinogenic factors, and finally transformed to HCC. It can explain why HCC only happens in ectopic liver and no lesion can be detected in the mother liver at the same time.

One of the diseases should be considered in differential diagnosis is hepatoid adenocarcinoma (HAC). HAC is a rare subtype of extra-hepatic adenocarcinoma, most frequently originate in the stomach, also arises in the ovaries, lungs, bladder, pancreas, and uterus,^[6]^ characterized by hepatic differentiation and an excessive production of AFP.^[7,8]^ Microscopic examination of HAC shows adenocarcinoma of common type intermingled with hepatoid foci.^[7,8]^ In the imaging, Chang et al^[9]^ observed the finding of arterial phase hyperenhancement and delayed phase washout pattern in these tumors. In this case, no lesion was detected in liver, stomach, ovaries, lungs, bladder, pancreas, or uterus. Besides, the serum AFP of this patient was in the normal level and his immunohistochemical results showed negative AFP expression in the tumor. MRI showed these masses presented with heterogeneous hyperenhancement in arterial phase, and more obvious hyperenhancement can be seen in portal venous and delayed phase, no washout of the contrast agent was detected. Compared with the characteristics of HAC, we ruled out the diagnosis of HAC.

Surgery resection seems to be one of the effective treatments for ectopic HCC, though it is detected with multiple tumors. In this case, the patient received surgery twice for tumor resection to attenuate the symptoms and improve his quality of life, with total survival time of 22 months. As the detailed mechanism of the development of ectopic HCC is not clear nowadays, further studies should be needed to clarify its mechanism and clinical feature, thus to improve the therapeutic effect.
